# P-125. Prospective Cohort Study of Children and Adolescents with Intracranial Collections of Infectious Etiology

**DOI:** 10.1093/ofid/ofaf695.352

**Published:** 2026-01-11

**Authors:** Manuel S Ordoñez, Mario A Bustos, Isabel Hurtado, Christian Rojas, Eduardo Lopez-Medina

**Affiliations:** Universidad del Valle, Cali, Colombia, Popayán, Cauca, Colombia; Universidad del Valle, Cali, Colombia, Popayán, Cauca, Colombia; Universidad del Valle, Cali, Colombia, Popayán, Cauca, Colombia; Universidad del Valle, Cali, Colombia, Popayán, Cauca, Colombia; Centro de Estudios en Infectología Pediátrica CEIP, Departamento de Pediatría, Universidad del Valle, Clínica Imbanaco, Grupo Quironsalud, Colombia., Cali, Valle del Cauca, Colombia

## Abstract

**Background:**

Infectious intracranial collections (IC) cause serious morbidity in children, especially in low- and middle-income countries due to high rates of predisposing infections. Early in the COVID-19 pandemic, IC cases appeared to increase, highlighting the need for ongoing surveillance and characterization.

**Figure:**
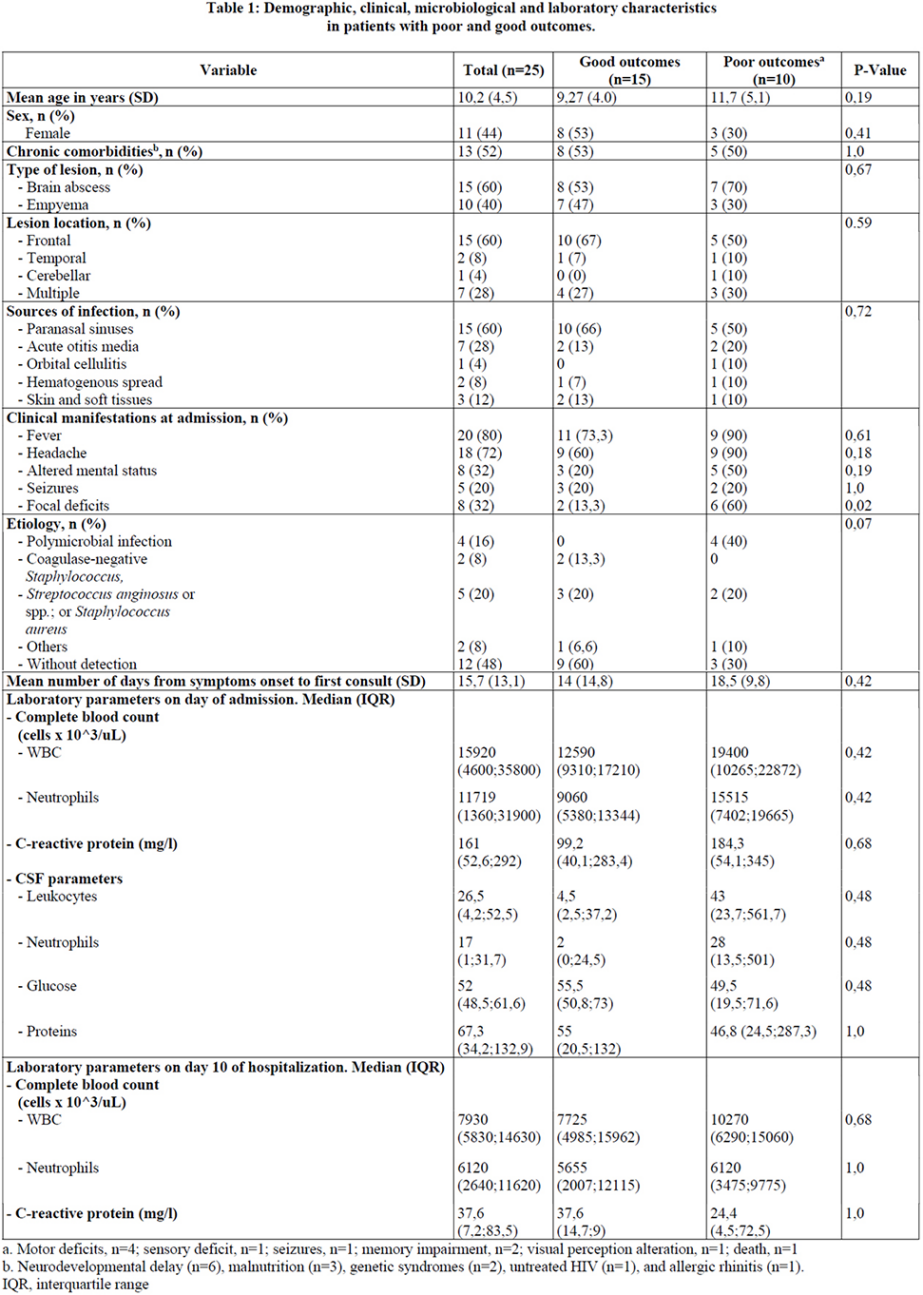

**Figure 1.**
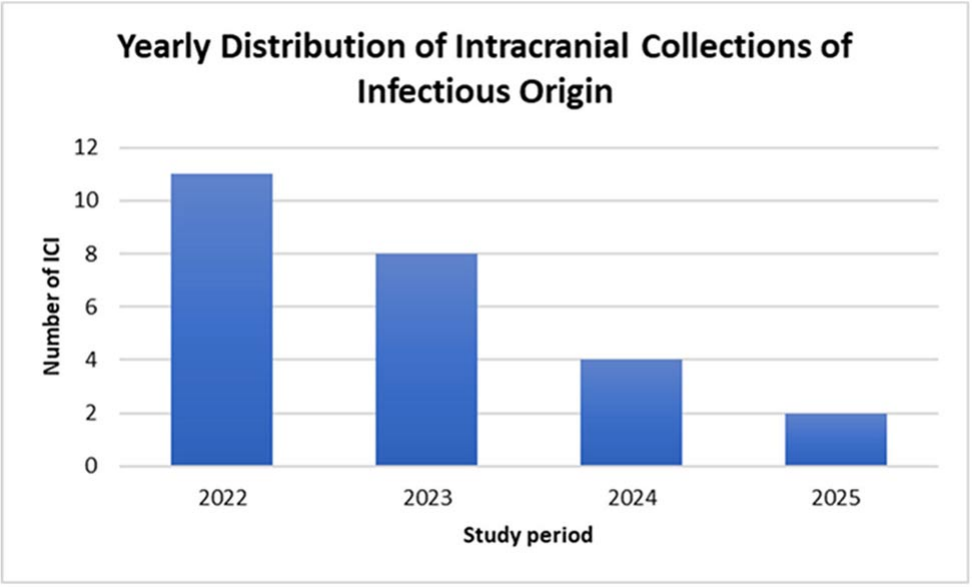
Yearly distribution of intracranial collections of infectious origin.

**Methods:**

Prospective cohort study of pediatric IC patients at two referral centers in Cali, Colombia (Jan 2022–Apr 2025). Cases were imaging-confirmed and excluded trauma- or device-related infections. Clinical, microbiological, and discharge data were collected to explore differences in patients with and without poor outcomes (death or neurologic sequelae). Ethics approval was obtained from both centers.

**Results:**

Twenty-five patients were diagnosed with IC, clustering after social measures relaxed in 2022 (Figure 1). Fourteen (56%) were male, median age 10.2 years (range 1–17), and 13 (52%) had chronic comorbidities (Table 1). Brain abscesses occurred in 15 (60%) (single: n=9; two: n=5; >five: n=1), 5 (20%) had only subdural empyema, 3 (12%) only epidural, and 2 (8%) had both. Median time from symptom onset to consult was 15.7 days (SD 13.1). Common symptoms were fever (80%, median 6 days, max 22) and headache (72%). Twenty (80%) underwent surgical drainage (45% reinterventions). Microbiological identification succeeded in 13, with 4 polymicrobial infections and 3 with *Streptococcus anginosus* or spp. Median hospital stay was 37.2 days (range 7–84). Fourteen required PICU admission (mean 3.1 days, max 15). Neurological sequelae occurred in 9, and 1 died. Poor outcomes were associated with focal neurological deficits at admission, polymicrobial infections, and higher CSF and systemic inflammation (Table 1). Median IV antibiotic duration was 28 days (range 7–56); most used were vancomycin, ceftriaxone, and metronidazole.

**Conclusion:**

IC in children carry a high burden and are often linked to poor outcomes. IC peaked early post-pandemic, likely due to increased viral circulation and secondary bacterial infections. Healthcare providers should educate families on seeking timely care for suspected bacterial respiratory infections while preventive measures are developed or improved against invasive respiratory bacteria.

**Disclosures:**

All Authors: No reported disclosures

